# The impact of antiphospholipid antibodies in Takayasu arteritis

**DOI:** 10.55730/1300-0144.5573

**Published:** 2022-11-01

**Authors:** Esra FIRAT ŞENTÜRK, Abdulsamet ERDEN, Alper SARI, Berkan ARMAĞAN, Levent KILIÇ, Umut KALYONCU, Ömer KARADAĞ, Şule APRAŞ BİLGEN, Sedat KİRAZ, İhsan ERTENLİ, Ali AKDOĞAN

**Affiliations:** 1 Department of Internal Medicine, Faculty of Medicine, Hacettepe University, Ankara, Turkey; 2 Division of Rheumatology, Department of Internal Medicine, Hacettepe University, Ankara, Turkey

**Keywords:** Takayasu arteritis, anti-beta 2 glycoprotein-1 antibodies, anti-cardiolipin antibody, lupus anticoagulant, antiphospholipid antibody syndrome

## Abstract

**Background/aim:**

The significance of antiphospholipid antibodies (aPL) is controversial in Takayasu arteritis (TA). This study was conducted to explore the frequency of aPL and their association with disease-related complications in TA.

**Materials and methods:**

This cross-sectional study was conducted to investigate the presence of anti-cardiolipin (aCL), anti-beta 2 glycoprotein-1(aβ2G1) antibodies, and lupus anticoagulant (LA) in TA patients. TA patients admitted to the Department of Rheumatology of Hacettepe University Faculty of Medicine between December 2015 and September 2016 who fulfilled the American College of Rheumatology (ACR) classification criteria for TA were consecutively enrolled in the study. Patients were grouped according to aPL positivity and compared in terms of disease manifestations, type of vascular involvement at diagnosis, and vascular complications/interventions attributable to TA.

**Results:**

Fifty-three TA (49 female) patients were enrolled in the study. We detected 9 (16.9%) patients with IgM and/or IgG aβ2G1 and/or LA positivity. There were no patients with positive aCL. All aβ2G1 titers were low. There were no differences in terms of symptoms, signs, type of vascular involvement, the number of patients with disease-related complications or vascular interventions/surgery between aPL (+) and aPL(−) groups (p > 0.05 for all). The number of patients with thrombotic lesions was similar between the groups (p > 0.05). There were no patients with a history of venous thrombosis or on anticoagulant treatment in the aPL(+) group. Only 1 patient with IgM aβ2G1 (+) had a history of pregnancy loss.

**Conclusion:**

Our results indicate that aPL positivity is not rare in TA. On the other hand, all aPL titers were low and no differences were found in the frequency of disease-related complications between aPL(+) and aPL(−) patient groups. Only TA patients with atypical manifestations with high suspicion of aPL-related complications should be considered to be investigated for aPL.

## 1. Introduction

Takayasu arteritis (TA) is a granulomatous large vessel vasculitis characterized by the involvement of the aorta and its branches. The disease is more common in Asia than in western populations and mainly affects young women. TA is considered to be a slow-progressing disease and the 10-year survival rate has been reported to be over 85% [1]. However, TA has an unpredictable clinical course with frequent disease exacerbations. Vascular involvement in TA can cause permanent organ dysfunction, even death in a significant number of patients in the short term. Therefore, identifying poor prognostic factors is crucial in TA.

Antiphospholipid syndrome (APS) is an autoimmune disease characterized by recurrent thrombosis and pregnancy complications due to autoantibodies [2]. It is reasonable to think that the presence of antiphospholipid antibodies (aPL) may increase vascular damage in the course of a vasculitic disease such as TA due to their thrombotic effects. Although there were studies and case reports supporting the association of aPL and TA severity in the literature, the clinical importance of these antibodies in TA is still controversial [3–7]. This study was conducted to explore the frequency of aPL and its association with disease-related complications in TA.

## 2. Materials and methods

Patients with TA admitted to the Department of Rheumatology of Hacettepe University Faculty of Medicine between December 2015 and September 2016 and who fulfilled the American College of Rheumatology (ACR) classification criteria for TA were consecutively enrolled in this cross-sectional study [8]. Pregnant patients or patients aged (<18) years were excluded. Medical histories of all TA patients; including both the history of thrombotic events and the obstetric complications (for female patients) in detail were reevaluated by face-to-face interview. Complete physical examinations were performed in all subjects. Medical records of the patients were reviewed retrospectively. The type of vascular involvement and involved arteries of the patients at diagnosis were determined by using the reports of imaging modalities (computed tomography angiography (CTA) and/or magnetic resonance angiography (MRA) and/or conventional angiography) [9]. The presence or history of hypertension, retinopathy, cerebrovascular accident, transient ischemic attack, severe aortic regurgitation, aortic valve replacement, aneurysm formation, vascular interventions (angioplasty and/or stent implementation), and vascular surgery were considered as disease-related complications and data about these were recorded as well. TA patients were also grouped according to the presence or absence of major complications (hypertension and/or aneurysm and/or aortic regurgitation and/or retinopathy) [10]. TA patients with arterial thrombotic lesions were also noted. TA patients who received cyclophosphamide and/or biological agents at any time were considered as treatment-resistant cases or severe diseases [11,12]. A history of antiaggregant and/or anticoagulant use was also recorded.

All subjects gave written informed consent and the institutional ethical committee approved this study.

### 2.1. Autoantibody analysis

Blood samples for testing autoantibodies were collected on the day of the evaluation of TA patients for the study. Tests for IgM/G anti-cardiolipin antibodies (aCL) and IgM/G anti-beta 2 glycoprotein-1 antibodies (aβ2G1) were performed by using a standardized enzyme-linked immunosorbent assay (ELISA) (Orgentec Diagnostika ^®^515 and 521 commercial assays, respectively, ORGENTEC Diagnostika, Germany). The normal reference range for IgG/IgM aCL was <10 U/mL and <7 U/mL, respectively (range 0–100 U/mL, for both). The normal reference range for IgG/IgM aβ2G1was <5 U/mL (range 0–100 U/mL, for both). Screening for lupus anticoagulant (LA) was performed by using the diluted Russell’s viper venom time assay in accordance with standard methods (Siemens Healthcare Diagnostics^®^, Germany). Confirmatory tests used a combination of mixing studies and correction with the addition of phospholipids. A LA ratio of 1.2 was considered to be positive (low positive: 1.2–1.5, medium positive 1.5–2.0, high positive >2.0).

### 2.2. Statistical analysis

Statistical analysis was performed using the Statistical Package for the Social Sciences software (version 21.0; IBM Corporation, Armonk, NY, USA). Unless indicated otherwise, values are expressed as mean ± SD. Fisher’s exact and chi-square tests were used for the comparison of categorical variables or percentages. Student t-test and Mann-Whitney U test were used to compare parameters among the groups whenever appropriate. A p-value of less than 0.05 was considered statistically significant.

## 3. Results

We included 53 TA (49 female) patients. The mean age of the TA patients was 40.8 ± 12.7 years. The mean age of the patients at diagnosis was 33.3 ± 11.4 years and the median disease duration was 48 months (interquartile range (IQR); 30.75–108.0 months). Constitutional symptoms (fever and/or fatigue and/or weight loss), extremity claudication, dyspnea, carotidynia, and palpitation were the most common complaints at the time of diagnosis in TA patients with the rate of 92.2% (n = 47), 64.7% (n = 33), 43.1% (n = 22), 31.4% (n = 16), 31.4% (n = 16), respectively. The age of symptoms onset was <40 years in all subjects. Decreased brachial artery pulses, bruits over subclavian arteries or aorta, and the blood pressure difference between the arms, were present in 49.1% (n = 26), 75.5% (n = 40), and 17.1% (n = 9) of patients, respectively. We detected 9 TA (16.9%) patients with IgM/G aβ2G1 and/or LA positivity. There were no patients with positive aCL. Of the 9 antibody positive patients; 7 had positive IgM aβ2G1, 3 had IgG aβ2G1 and 1 had positive LA. One had both positive IgM and IgG aβ2G1. Additionally, one had both IgM aβ2G1 and LA (Table 1). The mean serum level of IgM aβ2G1 was 6.6 ± 1.3 U/mL in antibody positive patients. The IgG aβ2G1serum levels of 3 patients were 5.0 U/mL, 7.2 U/mL, and 6.4 U/mL. Detected LA levels were considered as borderline positive with a ratio of 1.2. There were no patients with the infectious disease when blood samples were collected for antibody testing.

Demographic, clinical features and types of vascular involvement of aPL(+) and aPL(−) patients are represented in Table 2. There were no differences among the aPL(+) and aPL(−) patient groups in terms of age and gender. The median disease duration in the aPL(+) group was longer than the aPL (−) group (p = 0.02). The frequencies of the symptoms and signs were similar between two groups. The most frequent vascular involvement type was type V in both aPL(+) and aPL(−) groups. There were no differences in types of vascular involvement between the two groups (Table 2).

### 3.1. Complications

Among 52 TA patients (1 patient excluded from the analysis because of insufficient data) a total of 45 complications occurred in 28 patients during their follow-up. The rates of disease-related complications including vascular interventions or vascular surgery were not different between the aPL(+) and aPL(−) patients (p > 0.05 for all) (Table 3). No difference was found between the number of patients with complications or major complications (hypertension, aneurysm, aortic regurgitation, and retinopathy) between the groups (p > 0.05 for all). Forty-five patients were evaluated at least once by transthoracic echocardiography during their follow-up. Three patients had pulmonary hypertension (systolic pulmonary arterial pressure >40 mmHg). There was one patient with pulmonary hypertension in the aPL(+) and 2 in the aPL(−) group.

### 3.2. Immunosuppressive treatment

All TA patients were treated with steroids and immunosuppressive agents except one. The initial immunosuppressive agents were as follows; methotrexate in 29 (54.7%), azathioprine in 13 (24.5%), cyclophosphamide in 9 (16.9%), and mycophenolate mofetil in 1 (1.8%). One (1.8%) patient was treated initially with steroids and tocilizumab and was followed up with the diagnosis of ankylosing spondylitis, and was already on tumor necrosis factor alpha antagonist. During the follow-up period, a total of 15 (28.3%) patients were treated with cyclophosphamide and 16 (30.2%) patients with biologic agents. The number of patients treated with cyclophosphamide and/or biologic agents was not different between aPL (+) and aPL (−) groups (p = 0.29) (Table 4). There were 38 (71.6%) patients who were on low-dose acetylsalicylic acid. There was no difference between aPL(+) and aPL(−) patients who were on low-dose acetylsalicylic acid (77.7% vs. 70.4%; p = 1.00).

### 3.3. Thrombotic events and pregnancy loss

There were 9 patients with thrombotic lesions. All had systemic arterial thrombotic lesions except one who had documented pulmonary arterial thrombosis and pulmonary arterial involvement. This patient with pulmonary thrombosis was aPL (−). There was no difference in the number of patients with thrombotic lesions between aPL (−) and aPL (+) groups (6/42 vs. 3/9; p = 0.18). None of the aPL (+) patients had venous thrombotic events in their disease course. There were 4 patients who were on anticoagulant treatment (1 for atrial fibrillation, 1 for aortic valve replacement, 1 for immobilization due to severe pulmonary hypertension, and 1 for after bypass surgery). None of the patients who were on anticoagulant treatment were aPL (+).

Pregnancy loss occurred in 5 among 73 pregnancies in 49 female TA patients. Three of 5 pregnancy losses occurred after TA diagnosis and 4 of 5 pregnancy losses occurred >10 weeks of gestation. There were no patients with a history of recurrent pregnancy loss. There was only one pregnancy loss in aPL(+) TA patients. This patient was aCL IgM (+) and had a pregnancy loss >10 weeks of gestation. Only 3 (6.1%) patients stated that they had HT during pregnancy.

## 4. Discussion

In this study, aPLs were detected in 16.9% of the TA patients. All the antibody positive patients had IgM and/or IgG aβ2G1, there was only one patient with a positive LA test and there were no aCL positive patients. Serum concentrations of the antibodies were low in all patients with aβ2G1 positivity.

There are few studies in the literature investigating the frequency of aPL in TA patients. Most of those studies were cross-sectional and had a limited number of patients; they were conducted with 22, 28, 21, 19, and 47 TA patients, respectively [4,7,13–15]. The frequency of aPL was reported between 0% and 45%. Only in one study antibodies were evaluated more than once during the disease course and the frequency of persistent aPL positivity was found to be 45% (10/22 patients). In the same study, aPL were detected only once during the disease course in 7 of the remaining 12 patients [4]. Not all the aPL were evaluated in all studies. In our study, the frequency of aPL was 16.9% which was compatible with the literature. All the antibody positive patients had IgM and/or IgG aβ2G1 except one with LA positivity. Our results were similar to the Mexican study where the authors reported that there were no patients with positive aCL but detected low titers of IgM or IgG aβ2G1 in 39.2% (11/28 patients) of their TA patients [7].

The clinical significance of the presence of aPL in the course TA is controversial. Although severe cases with TA and aPL had been reported, the thrombotic events in these patients were usually detected in the arterial circulation, and the number of cases with venous thrombosis or recurrent pregnancy losses, which are more typical for APL, were low. A venous thrombotic event (in 3 patients) or recurrent pregnancy (in 4 patients) loss was only present in 7 of the 19 reported cases in the literature [5,6,16–34]. In studies where aPL were screened more systematically, it has been suggested that aPL in TA patients were associated with disease activity or an increased number of vascular interventions [3,4]. On the other hand, there were studies that reported no clinical association with aPL in TA [7,13–15]. Moreover, antibody titers were usually low in all of those studies; which were not enough to fulfill Sapporo classification criteria for definite antiphospholipid syndrome [2,4,7,15,35]. In the study where authors suggested an association between aPL and more frequent vascular interventions; there was only 1 out of 10 who met Sapporo criteria, in the aPL(+) group [4,35]. In our study, there were no differences in the symptoms, signs, type of vascular involvement, and number of patients with complications including vascular interventions or surgery between aPL(+) and aPL() groups. Moreover, the number of patients treated with cyclophosphamide and/or biological agents was not different between the groups. This result suggests that the rate of severe or treatment-resistant cases is similar in both groups. Therefore, it is hard to conclude that the presence of aPL is associated with a severe TA course according to our results.

Not all the aPL are pathogenic and they can be detected in several conditions such as infectious diseases, inflammatory disorders, malignancies, and usage of particular drugs [2]. The antibodies that do not cause thrombosis are usually of IgM type and their titers are low [2,36]. In this study, most of the patients had IgM type aβ2G1 and all the antibody titers were low. Therefore, the antibodies we detected might be nonpathogenic and it could be an explanation for not observing an association between aPL and TA manifestations in our study.

The number of patients with thrombotic lesions was similar between aPL(+) and aPL(−) patient groups in our study. All the thrombotic lesions were on the systemic arterial circulation except one in pulmonary arteries. The patient who had pulmonary thrombosis was aPL(−) and had severe pulmonary artery involvement. Moreover, there were no aPL(+) patients with a history of venous thrombosis or on anticoagulant treatment for thrombotic lesions. There was only one patient who had a >10-week pregnancy loss with low titer IgM aβ2G1. Recurrent venous thrombosis and pregnancy loss are the most common manifestations of APS [2]. Therefore, our findings regarding the absence or rarity of venous thrombosis and pregnancy loss, which were unexpected for the presence of APS; might support the nonpathogenic character of these antibodies in TA patients who are aPL(+). Actually, the presence of TA is a satisfactory explanation for the development of arterial thrombosis alone. Vascular damage, blood flow disturbances due to aneurysms or narrowed arteries, and prothrombotic state can all contribute to the development of arterial thrombosis in TA patients [37,38]. In situ thrombosis can occur in pulmonary arteries during the disease course [39].

In this cross-sectional study, we tested patients for aPL only once and had no control group. However, we tested the antibodies with well-known, commonly used methods in the context of high clinical correlations. In addition, as all the antibody titers of our patients were low, there was no need for a second test except for the LA positive patient. On the other hand, although they are unlikely to affect the aPL titers, it should be kept in mind that most of our patients were on immunosuppressive therapy. Differences in clinical approach to TA patients between the countries may have affected the number of vascular interventions and surgeries performed. Although we evaluated more patients compared to similar studies in literature, the limited number of the patients tested and retrospective data collection may have affected our results. Lastly, we did not evaluate TA disease activities which may have an impact on antibody titers.

In conclusion, our results indicate that aPL positivity is not rare in TA. On the other hand, all aPL titers were low and no difference was found in the frequency of arterial thrombotic lesions or disease-related complications between aPL(+) and aPL(−) groups. There were no patients with a history of venous thrombosis in aPL(+) group. We do not suggest routine evaluation of TA patients for the presence of aPL. We suggest performing these tests only in TA patients with atypical manifestations highly suspected of aPL-related complications.

## Figures and Tables

**Table 1:** Detected antiphospholipid antibodies in Takayasu arteritis patients.

Anti-beta 2 glycoprotein-1 IgM (+) patients, n (%)	7 (13.2)*
Anti-beta 2 glycoprotein-1 IgG (+) patients, n (%)	3 (5.7)*
Lupus anticoagulant (+) patients, n (%)	1 (1.9)**
Anti-cardiolipin IgM (+) patients, n (%)	0 (0.0)
Anti-cardiolipin IgG (+) patients, n (%)	0 (0.0)
Total number of patients with any positive test results, n (%)	9 (16.9)
Anti-beta 2 glycoprotein-1 IgM concentration (U/ml), median (IQR)	5.7 (5.3–7.9)
Anti-beta 2 glycoprotein-1 IgG concentration (U/ml), median (IQR)	6.8 (6.4–7.2)

* One patient had both positive IgG and IgM anti beta 2 glycoprotein-1 antibodies.

** One patient had both IgM anti beta 2 glycoprotein-1antibody and Lupus anticoagulant test positivity.

**Table 2:** Demographic, clinical features, and types of vascular involvement in aPL(+) and aPL(−) TA patients.

	aPL(+) TA patients, (n = 9)	aPL(−) TA patients, (n = 44)
**Gender (female/male)**	7/2	42/2
**Age (years** ± SD**)**	42.5 ± 14.7	40.4 ± 12.4
**Age at diagnosis ( years** ± SD**)**	30.9 ± 12.8	33.8 ±10.8
**Disease duration, months (IQR)**	108 (66–240)	48 (24–84)*
**Symptoms and signs at diagnosis, n (%)** **		
Constitutional symptoms	8 (88.8)	39 (92.8)
Extremity claudication	5 (55.5)	28 (66.6)
Dyspnea	5 (55.5)	17 (40.4)
Carotidynia	4 (44.4)	12 (28.5)
Palpitation	4 (44.4)	12 (28.5)
Decreased brachial artery pulses	5 (55.5)	21 (50.0)
Bruits over subclavian or aorta	6 (66.6)	34 (80.9)
Blood pressure difference between the arms	0 (0.0)	9 (21.4)
**Type of vascular involvement at diagnosis, n (%)**		
Type I	1 (11.1)	14 (31.8)
Type IIa	1 (11.1)	4 (9.1)
Type IIb	0 (0.0)	5 (11.4)
Type III	0(0.0)	1 (2.3)
Type IV	1(11.1)	0 (0.0)
Type V	6 (66.7)	20 (45.5)
**Coronary arteries**	1 (11.1)	2 (4.5)
**Pulmonary arteries**	2 (22.2)	7 (15.9)

p > 0.05 for all except

* p = 0.02,

** data is available for 51 patients

aPL: antiphospholipid antibodies, TA: Takayasu arteritis, IQR: interquartile range

**Table 3:** Frequency of disease-related complications in aPL(+) and aPL(−) TA patients.

	aPL(+) TA patients, (n = 9)	aPL(−) TA patients, (n = 43)
Total number of complications, n (%)	7 (77.7)	38 (88.3)
Cerebrovascular accident, n (%)	1 (11.1)	3 (6.9)
Transient ischemic attack, n (%)	0 (0.0)	3 (6.9)
Severe aort regurtation, n (%)	0 (0.0)	3 (6.9)
Aortic valve replacement, n (%)	0 (0.0)	1 (2.3)
Angioplasty or stent, n (%)	0 (0.0)	4 (9.3)
Bypass surgery, n (%)	2 (22.2)	2 (4.6)
Aneurysm, n (%)	0 (0.0)	8 (18.6)
Hypertension, n (%)	4 (44.4)	13 (30.2)
Retinopathy, n (%)	0 (0.0)	1 (2.3)
Number of patient with complications, n (%)	6 (66.6)	22 (51.1)
Number of patients with major complication*, n (%)	4 (44.4)	20 (46.5)

p > 0.05 for all

* Hypertension and/or aneurysm and/or aort regurgitation and/or retinopathy

** Data is available for 52 patients

aPL: antiphospholipid antibodies, TA: Takayasu arteritis

**Table 4:** Patients treated with cyclophosphamide or biologic agents in aPL(+) and aPL(−) TA patients.

	aPL(+) TA patients, (n = 9)	aPL(−) TA patients, (n = 44)	p
Neither cyclophosphamide nor biologic agent*, n (%)	3 (33.3)	24 (54.5)	0.29
Cyclophosphamide, n (%)	3 (33.3)	12 (27.2)	0.70
Biologic agent*, n (%)	4 (44.4)	12 (27.2)	0.43
Cyclophosphamide and/*or* biologic agent*, n (%)	6 (66.6)	20 (45.4)	0.29

* Tumor necrosis factor-α antagonist and/or interleukin-6 receptor antagonist

aPL: antiphospholipid antibodies, TA: Takayasu arteritis

## References

[1] De Souza AWS De Carvalho JF Diagnostic and classification criteria of Takayasu arteritis Journal of Autoimmunity 2014 48 79 83 10.1016/j.jaut.2014.01.012 24461381

[2] Cervera R Asherson RA Clinical and epidemiological aspects in the antiphospholipid syndrome Immunobiology 2003 207 1 5 11 10.1078/0171-2985-00213 12638896

[3] Wang H Ma J Wu Q Luo X Chen Z Circulating B lymphocytes producing autoantibodies to endothelial cells play a role in the pathogenesis of Takayasu arteritis Journal of Vascular Surgery 2011 53 1 174 180 10.1016/j.jvs.2010.06.173 20832232

[4] Jordan NP Bezanahary H D’Cruz DP Increased risk of vascular complications in Takayasu’s arteritis patients with positive lupus anticoagulant Scandinavian Journal of Rheumatology 2015 44 3 211 214 10.3109/03009742.2014.964305 25438797

[5] Gerede DM Yüksel B Tutar E Küçükşahin O Uzun C Spontaneous Coronary Artery Dissection in a Male Patient with Takayasu’s Arteritis and Antiphospholipid Antibody Syndrome Case Reports in Rheumatology 2013 2013 10.1155/2013/272963 PMC3727182 23956914

[6] Caso V Paciaroni M Parnetti L Cardaioli G Biscarini L Stroke related to carotid artery dissection in a young patient with Takayasu arteritis, systemic lupus erythematosus and antiphospholipid antibody syndrome Cerebrovascular Diseases 2002 13 1 67 69 10.1159/000047749 11810014

[7] Nava A Senecal JL Banales JL Raymond I Reyes PA Absence of antiphospholipid/co-factor antibodies in Takayasu arteritis International Journal of Cardiology 2000 75 S99 S104 10.1016/s0167-5273(00)00177-7 10980345

[8] Arend WP Michel BA Bloch DA Hunder GG Calabrese LH The American College of Rheumatology 1990 criteria for the classification of Takayasu arteritis Arthritis & Rheumatism 1990 33 8 1129 1134 10.1186/s13075-021-02675-9 1975175

[9] Hata A Noda M Moriwaki R Numano F Angiographic findings of Takayasu arteritis: a new classification International Journal of Cardiology 1996 54 S155 S163 10.1016/s0167-5273(96)02813-6 9119518

[10] Ishikawa K Natural history and classification of occlusive thromboaortopathy (Takayasu’s disease) Circulation 1978 57 1 27 35 10.1161/01.cir.57.1.27 21760

[11] Keser G Direskeneli H Aksu K Management of Takayasu arteritis: a systematic review Rheumatology (Oxford) 2014 53 5 793 801 10.1093/rheumatology/ket320 24097290

[12] Ohigashi H Tamura N Ebana Y Harigai M Maejima Y Effects of immunosuppressive and biological agents on refractory Takayasu arteritis patients unresponsive to glucocorticoid treatment Journal of Cardiology 2017 69 5 774 778 10.1016/j.jjcc.2016.07.009 27567177

[13] Girona E Amigo MC Izaguirre R Bañales JL Reyes PA Arteritis deTakayasu; IV. Ausencia de autoanticuerpos y anomalías de la coagulación/fibrinolisis Revista de Investigacion Clinica 1993 45 3 241 246 (in Spanish) 8105524

[14] Eichhorn J Sima D Thiele B Lindschau C Turowski A Anti-endothelial cell antibodies in Takayasu arteritis Circulation 1996 94 10 2396 2401 10.1161/01.cir.94.10.2396 8921779

[15] Park MC Park YB Jung SY Lee KH Lee SK Anti-endothelial cell antibodies and antiphospholipid antibodies in Takayasu’s arteritis: correlations of their titers and isotype distributions with disease activity Clinical and Experimental Rheumatology 2006 24 2 S10 S16 16859589

[16] Couture P Chazal T Rosso C Haroche J Léger A Cerebrovascular events in Takayasu arteritis: a multicenter case-controlled study Journal of Neurology 2018 265 4 757 763 10.1007/s00415-018-8744-8 29392458

[17] Santacruz JC Londoño JD Panqueva U Cuervo F Aortitis: An Unusual Inflammatory Complication of Systemic Lupus Erythematosus Cureus 2021 13 9 10.7759/cureus.18028 PMC8520493 34671519

[18] Yang A Nayeemuddin M Prasad B Takayasu’s arteritis and primary antiphospholipid syndrome presenting as hypertensive urgency Case Reports 2016 2016 bcr2015211752 10.1136/bcr-2015-211752 PMC4735419 26783006

[19] Fukui S Hirota S Iwamoto N Karata H Kawakami A Takayasu Arteritis With Antiphosphatidylserine /Prothrombin Antibody-Positive Antiphospholipid Syndrome: Case Report and Literature Review Medicine (Baltimore) 2015 94 51 e2345 10.1097/MD.0000000000002345 26705229 PMC4697995

[20] Laczik R Galajda Z Dér H Végh J Kerekes G Cardiac manifestations in a young man with Takayasu arteritis associated with antiphospholipid syndrome The Israel Medical Association Journal: IMAJ 2012 14 12 768 770 23393717

[21] Dhaon P Das SK Saran RK Parihar A Is aorto-arteritis a manifestation of primary antiphospholipid antibody syndrome? Lupus 2011 20 14 1554 1556 10.1177/0961203311412414 21846694

[22] Gupta S Chhabra P Gupta N Aggarwal P Recurrent first-trimester abortion in a young female: Rare presentation of Takayasu arteritis Journal of Family Medicine and Primary Care 2016 5 3 719 721 10.4103/2249-4863.197291 PMC5290795 28217618

[23] Lee HJ Hwang JP Kim HS Takayasu arteritis and antiphospholipid antibody syndrome in an elderly woman The Korean Journal of Internal Medicine 2015 30 6 934 937 10.3904/kjim.2015.30.6.934 26552473 PMC4642027

[24] Chakrabarti N Chattopadhyay C Aortoarteritis with systemic lupus erythematosus and secondary antiphopholipid syndrome Indian Dermatology Online Journal 2012 3 1 66 68 10.4103/2229-5178.93483 23130270 PMC3481929

[25] Kaku B Higuchi T Kanaya H Horita Y Yamazaki T Usefulness of fluorine-18-fluorodeoxyglucose positron emission tomography in a patient with Takayasu’s arteritis associated with antiphospholipid syndrome International Heart Journal 2006 47 2 311 317 10.1536/ihj.47.311 16607057

[26] Santiago MB Paz O Rare association of antiphospholipid syndrome and Takayasu arteritis Clinical Rheumatology 2007 26 5 821 822 10.1007/s10067-006-0277-3 16575491

[27] Morović-Vergles J Takayasu’s arteritis associated with antiphospholipid antibodies Rheumatology International 2006 26 8 773 774 10.1007/s00296-005-0065-4 16231120

[28] Jishi AA Krishnan PR Almawi WY Takayasu arteritis with high titre of antiphospholipid antibodies and MTHFR Polymorphism Journal of Thrombosis and Thrombolysis 2005 20 1 47 50 10.1007/s11239-005-2459-2 16133896

[29] Jean-jaquet RS Takayasu’s arteritis and antiphospholipid antibody syndrome in pregnancy Philippine Obstetrical and Gynecological Society 1998 22 3 99 106 12179674

[30] Korzets Z Barenboim E Bernheim J Mekori Y Bernheim J Mesangioproliferative glomerulonephritis, antiphospholipid antibodies, and Takayasu’s arteritis--is there a link? Nephrology, dialysis, transplantation: official publication of the European Dialysis and Transplant Association-European Renal Association 1998 13 4 991 993 10.1093/ndt/13.4.991 9568865

[31] Yokoi K Hosoi E Akaike M Shigekiyo T Saito S Takayasu’s arteritis associated with antiphospholipid antibodies. Report of two cases Angiology 1996 47 3 315 319 10.1177/000331979604700317 8638879

[32] El-Reshaid K Varro J al-Duwairi Q Anim JT Takayasu’s arteritis in Kuwait The Journal of Tropical Medicine and Hygiene 1995 98 5 299 305 7563255

[33] Yokoi K Akaike M Shigekiyo T Saito S (An elderly patient with Takayasu’s arteritis associated with antiphospholipid antibodies) Nihon Ronen Igakkai Zasshi 1994 31 9 716 719 (In Japanese). 10.3143/geriatrics.31.716 7823407

[34] Saxe PA Altman RD Takayasu’s arteritis syndrome associated with systemic lupus erythematosus Seminars in Arthritis and Rheumatism 1992 21 5 295 305 10.1016/0049-0172(92)90023-7 1351317

[35] Wilson WA Gharavi AE Koike T Lockshin MD Branch DW International consensus statement on preliminary classification criteria for definite antiphospholipid syndrome: report of an international workshop Arthritis and Rheumatism 1999 42 7 1309 1311 10.1002/1529-0131(199907)42:7<1309::Aid-anr1>3.0.Co;2-f 10403256

[36] Amin NM Antiphospholipid syndromes in infectious diseases Hematology/Oncology Clinics of North America 2008 22 1 131 143 10.1016/j.hoc.2007.10.001 18207071

[37] Akazawa H Ikeda U Yamamoto K Kuroda T Shimada K Hypercoagulable state in patients with Takayasu’s arteritis Thrombosis and Haemostasis 1996 75 05 712 716 10.1055/s-0038-1650353 8725710

[38] Springer J Villa-Forte A Thrombosis in vasculitis Current Opinion in Rheumatology 2013 25 1 19 25 10.1097/BOR.0b013e32835ad3ca 23143223

[39] Narechania S Renapurkar R Heresi GA Mimickers of chronic thromboembolic pulmonary hypertension on imaging tests: a review Pulmonary Circulation 2020 10 1 2045894019882620 32257112 10.1177/2045894019882620 PMC7103595

